# Negative effects of tumor cell nitric oxide on anti-glioblastoma photodynamic therapy

**DOI:** 10.20517/2394-4722.2020.107

**Published:** 2020-12-24

**Authors:** Albert W. Girotti, Jonathan M. Fahey, Witold Korytowski

**Affiliations:** 1 Department of Biochemistry, Medical College of Wisconsin, Milwaukee, WI 53226, United States; 2 Department of Biophysics, Jagiellonian University, Krakow 30-387, Poland

**Keywords:** Glioblastoma, photodynamic therapy, nitric oxide, inducible NO synthase

## Abstract

Glioblastomas are highly aggressive brain tumors that can persist after exposure to conventional chemotherapy or radiotherapy. Nitric oxide (NO) produced by inducible NO synthase (iNOS/NOS2) in these tumors is known to foster malignant cell proliferation, migration, and invasion as well as resistance to chemo- and radiotherapy. Minimally invasive photodynamic therapy (PDT) sensitized by 5-aminolevulinic acid (ALA)-induced protoporphyrin IX (PpIX) is a highly effective anti-glioblastoma modality, but it is also subject to NO-mediated resistance. Studies by the authors have revealed that glioblastoma U87 and U251 cells use endogenous iNOS/NO to not only resist photokilling after an ALA/light challenge, but also to promote proliferation and migration/invasion of surviving cells. Stress-upregulated iNOS/NO was found to play a major role in these negative responses to PDT-like treatment. Our studies have revealed a tight network of upstream signaling events leading to iNOS induction in photostressed cells and transition to a more aggressive phenotype. These events include activation or upregulation of pro-survival/ pro-expansion effector proteins such as NF-κB, phosphoinositide-3-kinase (PI3K), protein kinase-B (Akt), p300, Survivin, and Brd4. In addition to this upstream signaling and its regulation, pharmacologic approaches for directly suppressing iNOS at its activity *vs.* transcriptional level are discussed. One highly effective agent in the latter category is bromodomain and extra-terminal (BET) inhibitor, JQ1, which was found to minimize iNOS upregulation in photostressed U87 cells. By acting similarly at the clinical level, a BET inhibitor such as JQ1 should markedly improve the efficacy of anti-glioblastoma PDT.

## INTRODUCTION

Glioblastoma, also known as glioblastoma multiforme (GBM), is classified as a grade IV glioma by the World Health Organization and is one of the most aggressive and persistent of all known human tumors^[1–3]^. The yearly incidence of glioblastoma in the United States is ∼3 per 100,000 individuals. Difficulties in distinguishing highly invasive malignant zones from normal brain tissue make tumor resection very challenging^[2,3]^. Glioblastomas are known to be resistant to most conventional interventions, including ionizing radiation or chemotherapy with drugs such as cisplatin and temozolomide^[4–6]^. Drug resistance can either be inherent or acquired during treatment^[7]^. Photodynamic therapy (PDT), which employs non-ionizing radiation, has several advantages over radiotherapy or chemotherapy, including the ability to often overcome resistance associated with these treatments^[8–10]^. Nevertheless, various forms of pre-existing or treatment-induced resistance also apply for PDT^[11,12]^. One important example pertains to nitric oxide (NO) generated by inducible nitric oxide synthase (iNOS) in PDT-challenged tumor cells. There is solid evidence for this mode of resistance in glioblastoma cells as well as several other human cancer lines, including breast, prostate, and melanoma^[13,14]^. In addition to this anti-PDT effect, iNOS/NO has been shown to stimulate proliferation, migration, and invasion of cells that survive a photodynamic challenge. In this review, we discuss findings such as these and their implications on anti-glioma PDT at the clinical level. Relevant topics include: (1) NO and its underlying role in tumor promotion/persistence; (2) basic principles of PDT and how it suppresses solid tumors; (3) iNOS/NO-mediated hyper-resistance to PDT and hyper-aggressiveness of surviving cells; (4) mechanism of iNOS/NO induction by PDT; (5) tumor expansion via PDT-induced bystander effects; and (6) pharmacologic approaches for limiting the negative effects of iNOS/NO after PDT. Much of this discussion is based on studies carried out in the authors’ laboratories.

Two key aspects of these studies distinguish them from most others dealing with the pro-tumor effects of iNOS/NO: (1) Rather than simply using unchallenged tumor cells, we applied an oxidative stress-based challenge, *viz.* PDT, and assessed how it was affected by endogenous iNOS/NO; and (2) We discovered that, in most cases, it was PDT-upregulated iNOS rather than pre-existing (constitutive) enzyme that generated sufficient NO to stimulate resistance and surviving cell aggressiveness. Better recognition of these negative responses to PDT is needed in advance of developing approaches for mitigating them and improving PDT efficacy. What we discuss here may also provide new insights into how iNOS/NO could impact anti-tumor chemotherapy or radiotherapy.

## NITRIC OXIDE: TUMOR-PROMOTING VERSUS TUMOR-SUPPRESSING EFFECTS

NO is a short-lived free radical molecule (τ < 2 s in H_2_O) that diffuses freely on its own in aqueous media and, similar to O_2_, can partition into hydrophobic environments such as cell membranes^[15,16]^. Naturally occurring NO is generated by three enzyme isoforms in the nitric oxide synthase family: neuronal (nNOS/ NOS_1_), inducible (iNOS/NOS_2_), and endothelial (eNOS/NOS_3_)^[17,18]^. Whereas nNOS and eNOS operate at low constitutive levels and require Ca^2+^ and calmodulin for optimal activity, iNOS can be induced to relatively high levels and does not require stimulatory Ca^2+^ or calmodulin^[18]^. All three enzymes catalyze the five-electron oxidation of L-arginine to L-citrulline and NO at the expense of NADPH and O_2_. NO is involved in many different normo- and pathophysiologic processes. For example, eNOS-derived NO at low steady state levels (1–10 nM) stimulates cyclic-GMP formation, leading to blood vessel relaxation and lowering of blood pressure. In contrast, iNOS-derived NO at much higher levels (≥ 1 μM), as produced by vascular macrophages in response to infection, is cytotoxic and potentially carcinogenic, e.g., by inducing DNA mutations^[19,20]^. NO itself may act thusly by binding to iron in iron-sulfur or heme proteins, but often does so after reacting with superoxide radical (O_2_^−^) to give peroxynitrite (ONOO^−^), a strong indiscriminate oxidant^[21]^. If generated chronically, ONOO^−^ may be carcinogenic, e.g., by causing tyrosine nitration or initiating lipid peroxidation^[21]^. On the other hand, for established tumors, ONOO^−^ can be cytotoxic, since such tumors are typically more sensitive to oxidative pressure than normal counterparts^[19,20]^. In many tumor cells, including glioma cells, NO can activate pro-survival signaling pathways by modifying effector proteins such as soluble guanyl cyclase (sGC), hypoxia-inducible fctor-1α (HIF-1α), extracellular signal-regulated kinases-1 and −2 (ERK-1/2), epidermal growth factor receptor (EGFR), or protein kinase-B (Akt) via phosphoinositide-3-kinase (PI3K)^[22–25]^. Such modification may occur via S-nitrosation of thiol groups on specific cysteine residues^[26]^. In this case, NO itself does not react usually, but rather some oxidized form of NO such as nitrosyl anhydride (N_2_O_3_) or a trans-nitrosating species such as S-nitroso-glutathione (GSNO)^[26–28]^. iNOS-derived NO from myeloid-derived suppressor cells (MDSCs) may also benefit malignant tumors by inactivating anti-tumor cytotoxic T-cells^[29]^. In this case, the cytotoxic agent is a strong NO-derived oxidant such as ONOO^−^. There is increasing evidence that endogenous NO at low levels (e.g., from tumor cells themselves or proximal vascular cells) can also increase tumor resistance to ionizing radiation or chemotherapeutic agents such as cisplatin and docetaxel^[30]^. This has been amply demonstrated for malignant gliomas, glioma stem cells (GSCs) in these tumors exhibiting much of this resistance^[31]^. Significant resistance to non-ionizing photodynamic therapy can also develop, which is discussed after basic principles of this treatment are described.

## ANTI-TUMOR PHOTODYNAMIC THERAPY: SOME BASIC PRINCIPLES

Photodynamic therapy (PDT) was introduced about 45 years ago as a novel means of selectively eradicating a variety of solid malignancies, many of which are refractory to conventional chemotherapy or radiotherapy^[32–34]^. PDT is a minimally invasive modality which typically exhibits little, if any, off-target cytotoxicity. Classical PDT consists of three operating components: (1) an administered photosensitizing agent (PS); (2) PS photoexcitation by non-ionizing radiation, typically in the far visible to near-infrared wavelength range; and (3) molecular oxygen^[32–34]^. For many tumors, including glioblastomas, light can be delivered interstitially via fiber optic networks, making this approach highly selective for the tumor target^[33,34]^. Without photoactivation, most PS are innocuous to tumor cells as well as normal cells, which distinguishes these PS from many chemotherapeutic agents, e.g., platinum-based drugs. In a common photodynamic reaction (Type II process), ground state PS is excited to a meta-stable singlet state, which crosses over to a longer-lived triplet excited state. The latter then transfers energy to ground-state O_2_, giving singlet molecular oxygen (^1^O_2_), a cytotoxic reactive oxygen species (ROS)^[33,34]^. For some PS, more complex electron or hydrogen transfer may occur (Type I process), resulting in formation of free radical or free radical-derived ROS, e.g., superoxide (O_2_^−^·), hydroxyl radical (HO·), and hydrogen peroxide (H_2_O_2_). Similar to ^1^O_2_, these ROS can kill tumor cells by oxidizing vital molecules (proteins, lipids, and nucleic acids) and activating death signaling pathways^[34]^. In 1995, Photofrin®, a hematoporphyrin oligomer, became the first PS to be FDA-approved for anti-tumor PDT, esophageal malignancies being treated initially^[32]^. Since then, PDT with Photofrin® and other PSs has been used to combat numerous other malignancies, including prostate, breast, cervical, head and neck, and brain (gliomas)^[33,34]^. PDT is now considered one of the most promising alternatives to radiotherapy and chemotherapy for treating highly aggressive brain malignancies such as glioblastoma^[8–10,35,36]^. One explanation for this pertains to distinct subcellular targets. PDT usually damages cytoplasmic organelles (mitochondria, lysosomes, and endoplasmic reticulum), whereas radiotherapy (X-rays and γ-rays) and chemotherapy (e.g., with platinum-based drugs) damage nuclear DNA^[37]^. As a result, any constitutive or acquired resistance to chemo- or radiotherapy may not apply when PDT is used. Moreover, PDT elicits a robust anti-tumor immune response, and this provides an additional advantage by eliminating cells that might withstand a PDT challenge^[38]^.

**Figure 1.**
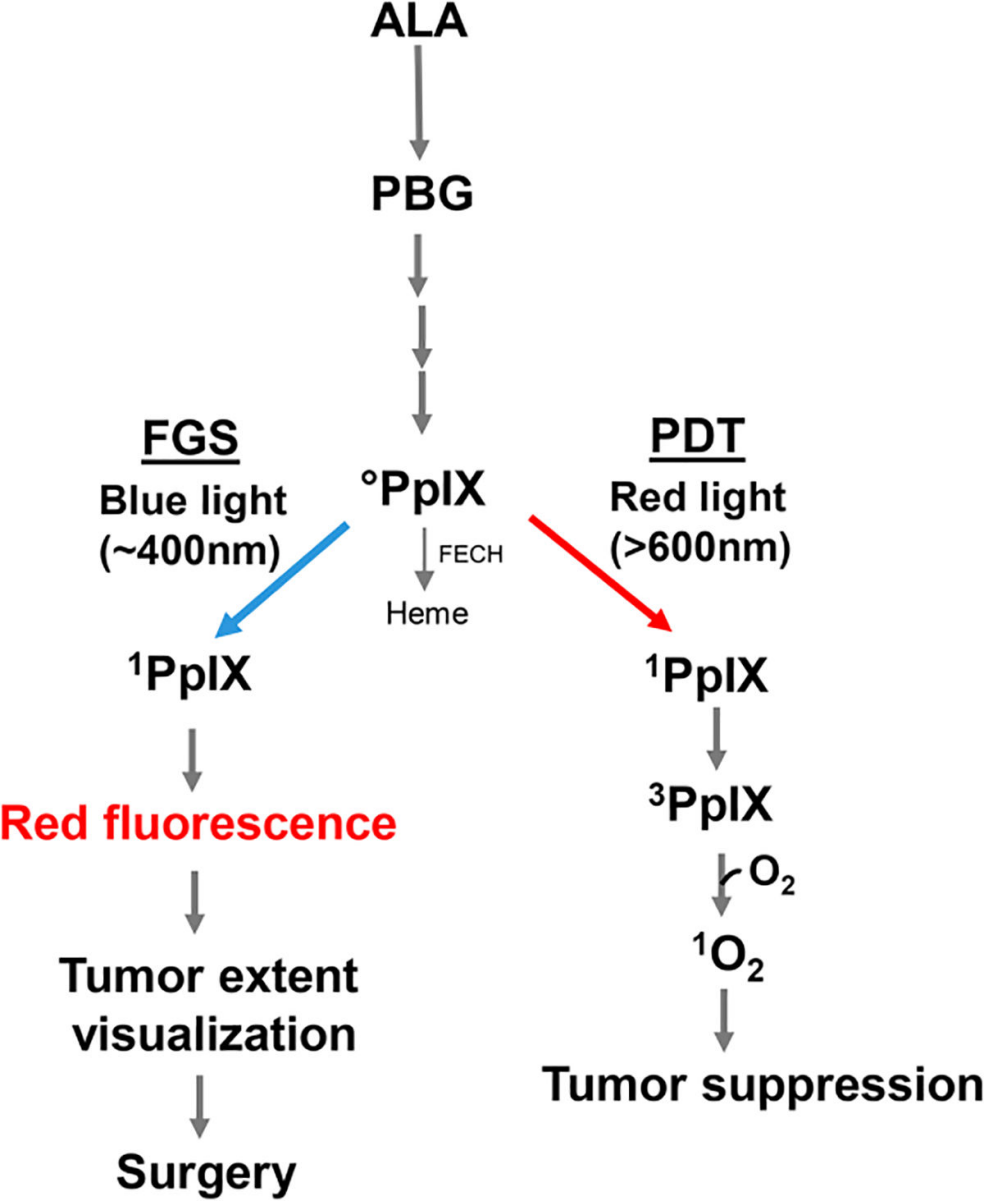
5-Aminolevulinic acid (ALA)-induced protoporphyrin IX (PpIX) via the heme biosynthetic pathway. Utilization of PpIX for photodynamic therapy (PDT) or fluorescence-guided surgery (FGS) is illustrated. PBG: porphobilinogen; FECH: ferrochelatase; ^0^PpIX: ground state porphyrin; ^1^PpIX: singlet excited state porphyrin; ^3^PpIX: triplet excited state porphyrin; ^1^O_2_: photogenerated singlet oxygen

Unlike Photofrin® and other PSs that are administered as such, pro-sensitizers have been developed which are converted to active PS after being administered. One important example is 5-aminolevulinic acid (ALA), which enters tumor cells via an amino acid transporter and is metabolized to active PS, protoporphyrin IX (PpIX), via the heme biosynthetic pathway [Figure 1], the PpIX accumulating initially in mitochondria^[39–41]^. This pathway is typically more active in malignant cells (e.g., glioblastomas) than normal counterparts, which accounts, at least in part, for the greater PDT susceptibility of the former. Moreover, unlike normal brain, malignant brain typically loses most of its blood-brain barrier function, allowing ALA access via the blood stream. In some tumors, including glioblastomas, this PpIX buildup is augmented by partial downregulation of ferrochelatase (FECH), the enzyme that inserts ferrous iron into PpIX to give heme^[40,41]^. In ALA-based PDT [Figure 1], relatively intense red light elevates ground state PpIX to a singlet excited state (^1^PpIX), much of which undergoes intersystem crossing to a long-lived triplet (^3^PpIX). Energy transfer from the latter to O_2_ gives ^1^O_2_, a non-radical ROS, whereas indirect reaction could give free radical ROS, as indicated above. In addition to sensitizing cytotoxic PDT reactions, ALA-induced PpIX can be used diagnostically to define tumor boundaries. In this case, low intensity blue light (∼400 nm) generates significant ^1^PpIX, which, upon decay to ground state, releases red fluorescent light. Many oncologists, particularly those treating difficult glioblastomas, have exploited this property for fluorescence-guided surgery (FGS), i.e., for clear demarcation of tumor boundaries before surgical resection [Figure 1]^[42]^. When applied carefully, using a surgical fluorescence microscope, FGS can greatly improve procedural accuracy by limiting inadvertent removal of non-tumor tissue^[42–44]^. Thus, ALA-induced PpIX has the advantage of serving as a surgical guide on the one hand and PDT sensitizer on the other hand. In addition to being used individually, FGS and PDT are often run sequentially, the latter to eradicate any residual tumor cells after the former is carried out^[44]^. Various pharmacologic approaches have been used for improving both FGS and PDT efficacy, e.g., FECH inhibitors or iron chelators^[44]^ to further elevate ALA-induced PpIX levels. ALA-based FGS and PDT are rapidly becoming the new standards of care for the management of malignant brain tumors.

## ANTAGONISTIC EFFECTS OF ENDOGENOUS NO IN GLIOBLASTOMA PDT MODELS

About 20 years ago, Henderson *et al.*^[45]^ and Korbelik *et al.*^[46]^, using various mouse syngeneic tumor models (e.g., RIF, SCCVII, and EMT6) and Photofrin® as PS, were the first to determine how endogenous NO might affect PDT efficacy *in vivo*. They showed that PDT cure rate could be significantly improved when NG-nitro-L-arginine (L-NAME), a non-specific inhibitor of NOS activity, was administered immediately after irradiation. A striking correlation was made between NO output and extent of improvement with L-NAME: tumors with the highest output responded best and those with the lowest output worst^[46]^. It was concluded that endogenous NO signaled for increased tumor resistance to PDT repression and did so in a NO dose-dependent manner. The L-NAME effects were principally attributed to NO’s vasodilatory effects acting in opposition to PDT’s known constrictive effects on the tumor microvasculature^[45,46]^. Follow-up studies by Reeves *et al.*^[47]^, using ALA-induced PpIX as PDT sensitizer for mouse RIF and EMT6 tumors, confirmed the above findings and again concluded that endogenous NO, by opposing vascular damage, can significantly increase tumor resistance to PDT. Although these studies^[45–47]^ and more recent ones by Rapozzi *et al.*^[48]^ clearly established that NO can antagonize PDT, several key questions were left largely unsettled, which include: (1) whether this NO is generated by tumor cells per se, proximal endothelial cells, macrophages, fibroblasts, or possibly all of these; (2) which NOS isoform plays a dominant role in any given tumor; (3) whether the NOS/NO in question acts at a pre-existing level or is upregulated in response to PDT stress; and (4) the signaling mechanisms involved in NOS expression and NO-induced resistance. Over the past ten years, the authors and lab colleagues have focused on these questions using various cancer cell lines, including glioblastoma lines. Key findings from this work are discussed below.

### Hyper-resistance imposed by photostress-upregulated iNOS/NO

**Figure 2.**
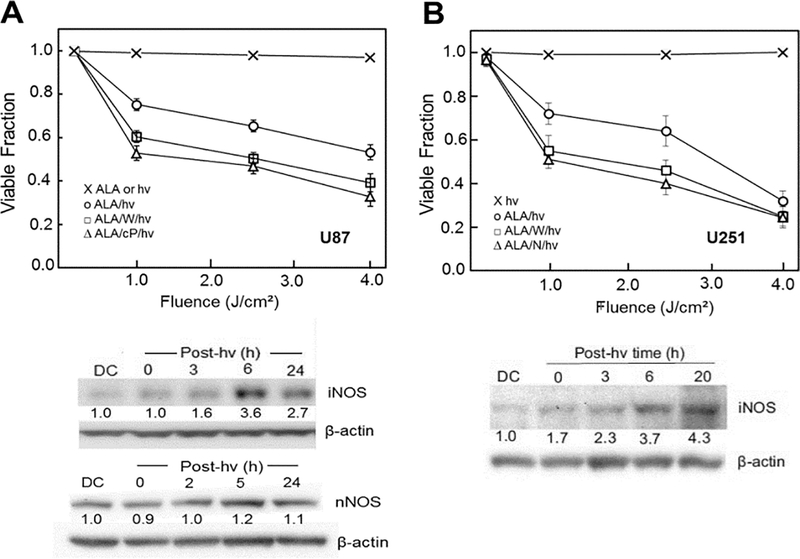
Viability loss of ALA/light-challenged glioblastoma cells and inhibition thereof by stress-induced iNOS/NO. Cells ∼ 60% confluency in serum-free medium were dark-incubated with 1 mM ALA for 30 min, switched to ALA-free medium, then irradiated with increasing fluences of broad-band visible light in the absence or presence of 25 μM 1400W (W), 1mM L-NAME (N), or 25 μM cPTIO (cP). ALA-only and light-only controls were run alongside. After treatment, cells were switched to serum-containing medium and after 20 h of dark incubation, assessed for viability by MTT assay or iNOS and nNOS status by Western blot analysis. A: U87 cells; B: U251 cells. Plotted values in (A) and (B) are means ± SEM (*n* = 3). Number below each NOS band is intergrated band Intensity relative to β-actin and normalized to the dark control (DC). ALA: 5-Aminolevulinic acid; PpIX: protoporphyrin IX; NO: nitric oxide; iNOS: inducible NO synthase; nNOS: neuronal NO synthase; post-hv: post-irradiation (Reproduced from Ref. 56, with permission)

As indicated above, PDT can often circumvent any innate or acquired tumor resistance to conventional chemotherapy or radiotherapy. It is now clear, however, that resistance mechanisms also exist for PDT, some of which are acquired during treatment. For example, there is evidence that activity of cytosolic ROS scavenging enzymes such as type-1 glutathione peroxidase and catalase are increased in lymphocytes subjected to a modest photodynamic challenge^[49]^. In addition, many cancer cell types, including glioblastomas, can export PpIX and other PS via the ABCG2 transporter, inhibition of which increases photosensitivity^[44,50]^. Another PDT resistance mechanism, which was discovered in the authors’ laboratory, involves NO generated specifically by tumor cell iNOS, particularly that which is upregulated in response to PDT stress^[51–55]^. This was demonstrated in recent experiments carried out on human glioblastoma U87-MG and U251-MG cells (henceforth referred to as U87 and U251)^[56]^. As shown in Figure 2A, U87 cells sensitized in mitochondria with ALA-induced PpIX were progressively inactivated after exposure to increasing fluences of broad-band visible light, 4 J/cm^2^, reducing the viable fraction by ∼45% 20 h after irradiation^[56]^. ALA alone or light alone was completely innocuous. When added before ALA/light treatment, 1400W (an enzyme inhibitor with a high specificity for iNOS) increased the extent of cell photokilling throughout, as did the NO scavenger, 2-(4-carboxyphenyl)-4,4,5,5-tetramethylimidazoline-1-oxyl-3-oxide (cPTIO) [Figure 2A]. Similar results were obtained with U251 cells [Figure 2B]. ALA/ light-induced U87 or U251 cell death occurred primarily via intrinsic (mitochondria-initiated) apoptosis, as assessed with Annexin V-fluorescein isothiocyanate (V-FITC), and this was substantially enhanced by 1400W or cPTIO, again consistent with iNOS/NO-imposed resistance^[56]^. Thus, it appeared that NOS-derived NO in U87 and U251 cells was acting cytoprotectively after a PDT-like challenge. When immunoblot analysis was used to assess iNOS status in these cells, it was found that the enzyme level increased progressively during post-irradiation incubation. After 6 h, it reached ∼4-times the basal level in U87 cells [Figure 2A] as well as U251 cells [Figure 2B]. As expected for this phenotype, U87 cells also expressed nNOS^[56]^, but, unlike iNOS, it was not upregulated after ALA/light treatment [Figure 2A]. Therefore, both glioblastoma cell types studied added significantly to their expressed iNOS the after a photodynamic challenge, and the resulting NO clearly enhanced their resistance to photokilling. Evidence for a large boost in NO steady state level was obtained by using the fluorescent probe diaminofluorescein-2-diacetate (DAF-2DA) which, after cell uptake and hydrolysis, detects NO via a byproduct such as N_2_O_3_^[57]^. Photostress-upregulated iNOS/NO has also been observed in human melanoma, breast, and prostate cancer lines, some of which, e.g., prostate PC3, boosted iNOS to much higher levels (8–10 folds) than evidenced in U87 or U251 cells^[53–55]^.

Fahey and Girotti^[58]^ recently extended the above *in vitro* findings to the *in vivo* level, using female immunodeficient (SCID) mice engrafted with breast MDA-MB-231 tumors. After intraperitoneal ALA administration, mouse tumors were irradiated, using a 633-nm Omnilux-Revive® LED source. Tumor growth in irradiated animals was significantly reduced compared with that in light-only controls over a 1–2-day period post-irradiation. However, an iNOS activity inhibitor (1400W or GW274150) in multiple doses (once daily over nine days) reduced growth much further, implying that iNOS/NO was stimulating tumor resistance to PDT^[58]^. For control animals irradiated without prior ALA treatment, 1400W had little (if any) effect on tumor growth, suggesting that pre-existing iNOS/NO had no significant protective effect^[58]^. Analysis of tumor samples after ALA-PDT revealed a striking ∼5-fold upregulation of iNOS protein over a low basal level, as well as a 1400W-inhibitable increase in NO-derived nitrite^[58]^. This was the first published *in vivo* evidence for iNOS upregulation by PDT and for increased resistance imposed by iNOS-derived NO. It should be emphasized that the bulk of this resistance was due to stress-upregulated iNOS/NO. This possibility has not been well recognized heretofore, either for PDT or other cancer therapies. Given that iNOS-generated NO is known to antagonize *in vivo* chemo/radiotherapy for glioblastoma^[24,25]^, it is likely that when evidence becomes available, it will also apply to *in vivo* PDT for glioblastoma, at least in an animal model.

### Hyper-aggressiveness of cells that survive PDT

**Figure 3.**
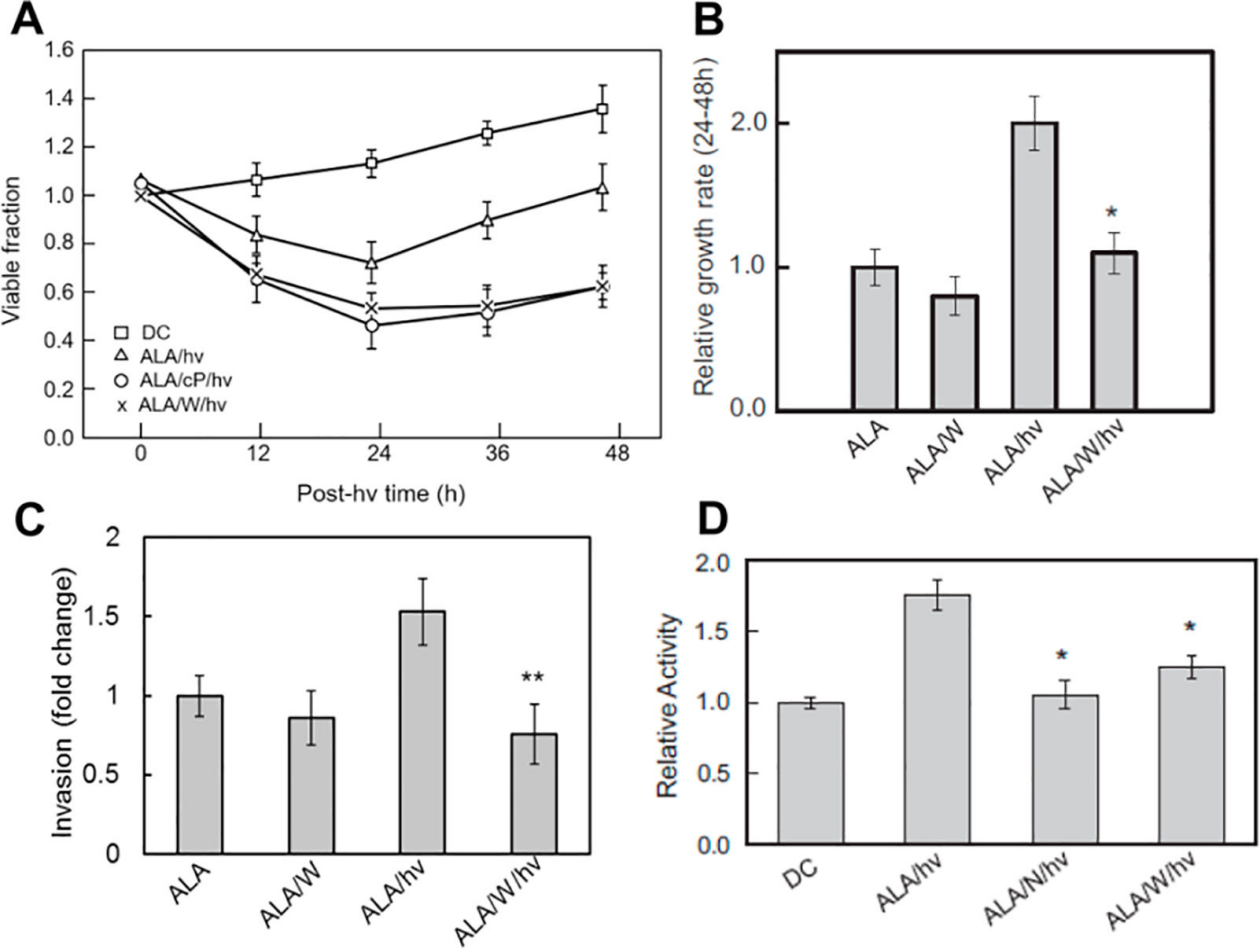
Enhanced proliferation and invasion rate of glioblastoma cells that survive ALA/light treatment: iNOS/NO dependency. U87 cells were sensitized with ALA-induced PpIX as described in Figure 2, exposed to a light fluence of ∼ 1 J/cm^2^, and then assessed for various post-irradiation parameters. 1400W(W), L-NAME (N), or cPTIO (cP) was either absent or present throughout. A: loss of viability (0–24 h post-hν) and subsequent proliferation of surviving cells (24–48 h post post-hν); DC: ALA-only dark control; B: surviving cell proliferation rate for selected conditions represented in panel (A); C: surviving cell invasiveness measured with a trans-well device; D: matrix metalloprotein-9 (MMP-9) activity measured by gelatin zymography 24 h after cells were ALA-light-treated. Plotted values are means ± SEM (*n* = 3); *P < 0.01 *vs.* ALA/hν (B); **P < 0.01 *vs.* ALA/hν (C); *P < 0.01 *vs.* ALA/hν (D). ALA: 5-Aminolevulinic acid; PpIX: protoporphyrin IX; NO: nitric oxide; iNOS: inducible NO synthase; post-hν: post-irradiation (Reproduced from Ref. 56 and 62, with permission)

When ALA/light-treated U87 cells *in vitro* were tracked beyond a 24 h post-irradiation point [*cf.* Figure 2], a striking observation was made, *viz.* that surviving cells (still attached) were proliferating more rapidly than controls over at least two additional days [Figure 3A]^[56,59]^. Similar to the viability loss over the first 24 h, the increase in proliferation rate was strongly attenuated by 1400W or cPTIO [Figure 3A]. In additional experiments, Fahey *et al.*^[56]^ showed that 1400W nearly nullified the ∼2-fold spurt in surviving cell proliferation, but had essentially no effect on proliferation of a dark (ALA-only) control [Figure 3B]. This suggested that preexisting iNOS/NO, unlike the upregulated forms, had little (if any) effect on cell division rate. Two additional manifestations of U87 hyper-aggressiveness were observed after an ALA/ light challenge: (1) accelerated migration, as assessed by a gap-closure (wound-healing) assay; and (2) accelerated invasion, as assessed by a trans-well assay involving cell traversal through an extracellular matrix (ECM)-like interface^[56]^. The large increase in migration rate (not shown) and invasion rate [Figure 3C] of photostressed cells was strongly blunted by 1400W, which, once again, had no significant effect on a dark control. As observed for proliferation, therefore, the more aggressive migratory and invasive properties of U87 cells that could withstand photostress were strongly dependent on upregulated iNOS/NO. Matrix metalloproteinases (MMPs) such as zinc-containing MMP-9 catalyze the degradation of collagen and other ECM components, and thus play a key role in cancer cell invasiveness and metastasis^[56]^. Innate migration and invasion of glioma cells is known to be promoted by MMP-9, which becomes activated by proteolytic cleavage of its exported precursor, pro-MMP-9^[60]^. Using in-gel zymography to measure the activity of externalized MMP-9 in ALA/light-stressed U87 cells, Fahey *et al.*^[56]^ found it to be ∼80% higher than that of dark controls [Figure 3D]. As shown, L-NAME and 1400W strongly inhibited this activation, pointing again to substantial iNOS/NO dependency. Immunoblot-assessed expression of three other proteins known to play important roles in glioblastoma aggressiveness was also 1400W-inhibitable in photostressed U87 cells: (1) downregulation of tissue-inhibitor of metalloproteinase-1 (TIMP-1); (2) upregulation of anti-apoptotic Survivin; and (3) upregulation of pro-metastatic S100A4^[56]^. Strong induction of S100A4 was the most remarkable of these because this protein was barely detectable in a dark control. Much remains to be learned about how NO modulated the expression of these effector proteins; however, the observed modulations are all consistent with the photostress responses shown in Figure 3.

## MECHANISMS OF INOS UPREGULATION AND NO-MEDIATED RESISTANCE IN PHOTOSTRESSED CELLS

**Figure 4.**
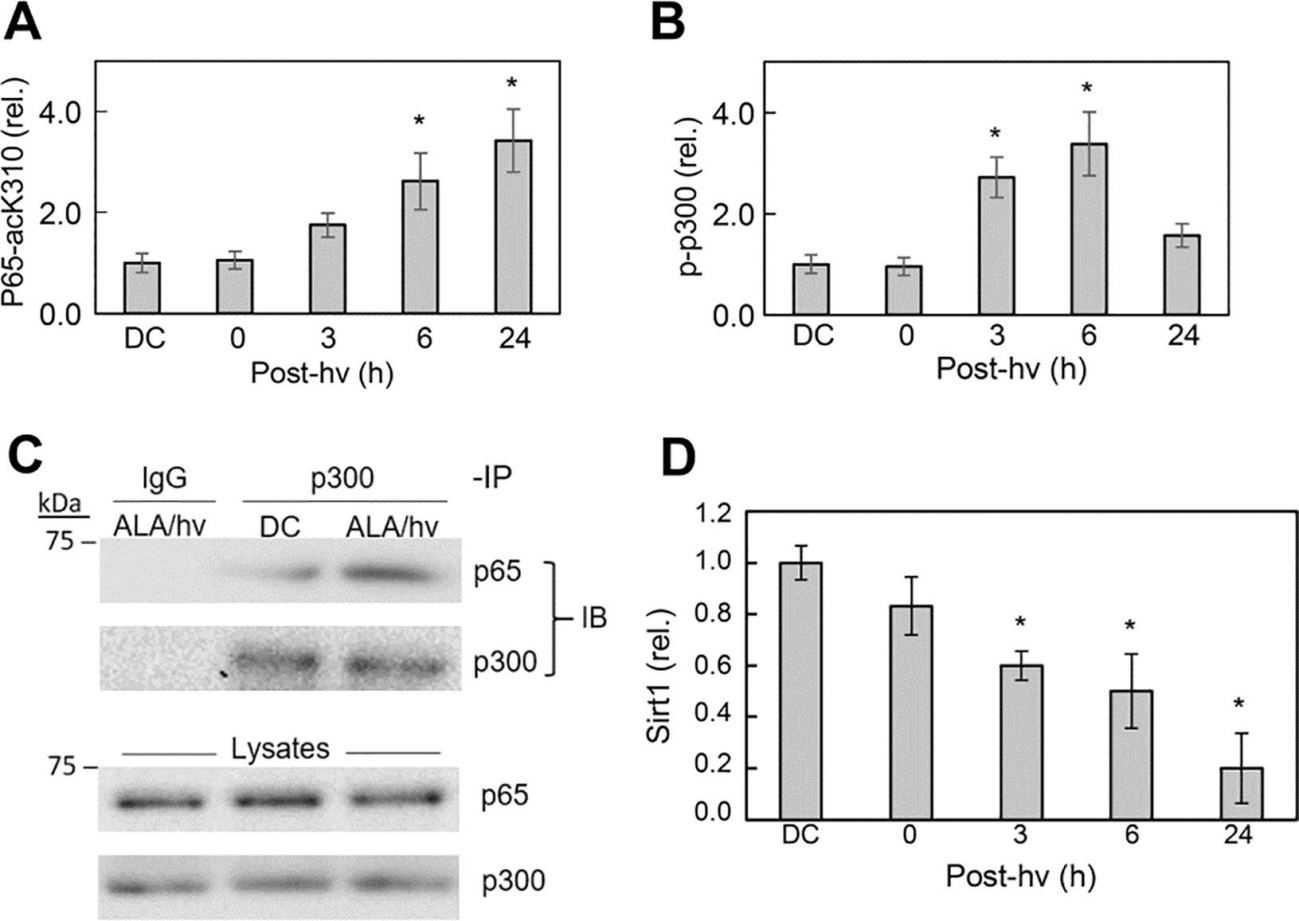
Some upstream evens that signal for greater iNOS transcription in ALA/light-treated glioblastoma U87 cells. A: post-irradiation upregulation of p65-ack310; DC: ALA-only dark control; B: post-irradiation upregulation of phosphorylation-activated p300 (p-p300); C: stimulation of p300 and p65 association in photostressed cells, as assessed by immunoprecipitation assay, using monoclonal p300 and p65 antibodies; D: time-dependent down-regulation of Sirt1 in photostressed cells, as assessed by immunoblot analysis. ALA: 5-Aminolevulinic acid; NO: nitric oxide; iNOS: inducible NO synthase; post-hν: post-irradiation; Sirt1: sirtuin 1 (Reproduced from Ref. 59, with permission)

**Figure 5.**
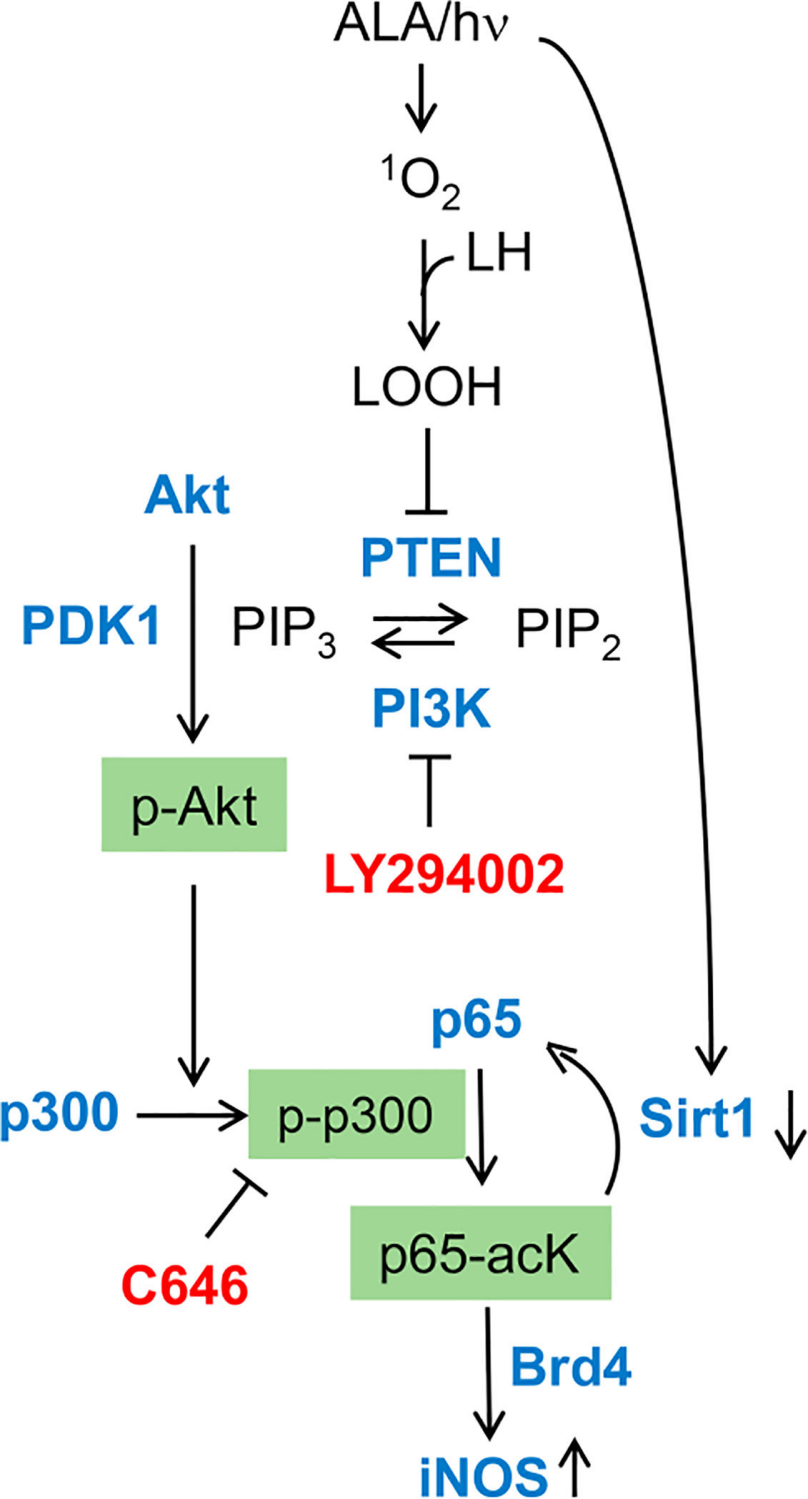
Photostress-induced upstream signaling events leading ultimately to iNOS transcriptional upregulation. Key effectors (LY294002, C646) and their protein targets. ALA: 5-Aminolevulinic acid; NO: nitric oxide; iNOS: inducible NO synthase; hν: irradiation; ^1^O_2_: photogenerated singlet oxygen; LOOH: hydroperoxide of mitochondrial membrane lipids; PTEN: phosphatase and tensin homologue; PIP3: phosphatidylisositol-3,4,5-triphosphate; PIP2: phosphatidylinositol 4, 5-bisphosphate; PI3K: phosphoinositide 3-kinase; PDK1: 3-Phosphoinositide-dependent protein kinase-1; p-Akt: phosphorylated Akt; p-p300: phosphorylation-activated p300; Sirt1: sirtuin 1; Brd4: bromodomain-containing protein 4; NO: nitric oxide; iNOS: inducible NO synthase (Reproduced from Ref. 59, with permission)

Regarding underlying mechanisms, most research to date has focused on how iNOS is upregulated by photodynamic stress rather than how the resulting NO signals for greater cell resistance to photokilling, although some headway has been made on the latter issue. Early studies on human breast COH-BR1 cells^[51–53]^ and more recent ones on glioblastoma U87 and U251 cells^[56,59]^ revealed that activation of transcription factor NF-κB is necessary for iNOS transcription in response to an ALA/light challenge. NF-κB activation may have been due to engagement of stress signaling elements IRE1 or PERK^[61]^. Our evidence indicated that NF-κB subunit p65/Rel A of ALA/light-treated U87 cells translocated from the cytosol to nucleus for initiation of iNOS transcription. Based on non-glioma studies by Huang *et al.*^[62]^, we postulated that acetylation of specific lysine residues in p65 was necessary for stimulating transcription. As supporting evidence, Fahey *et al.*^[63]^ showed that acetylation of lysine-310 (p65-acK310) increased progressively during post-irradiation incubation of U87 cells, reaching > 3-times the control level after 24 h [Figure 4A]. The rise in acK310 level was blocked by C646, an inhibitor of activated p300, confirming that the latter had catalyzed this acetylation^[63]^. The acetyltransferase p300 and its paralog CREB-binding protein (CBP) act as transcriptional co-activators for several tumor-promoting transcription factors^[64,65]^. p300 stimulates gene expression at promoter sites by catalyzing acetylation of specific lysine residues on histones or transcription factors such as NF-κB^[64]^. Therefore, we determined whether p300 is involved in p65-K310 acetylation and, if so, how photodynamic stress might affect p300 expression/activity. Immunoblot analysis revealed that photostress had no effect on overall p300 level relative to a dark control. As with p65-acK310 build-up, however [Figure 3A], there was a progressive increase in activated p300, i.e., Ser-1834-phosphorylated enzyme (p-p300), over at least a 6-h post-hν period [Figure 4B]. Moreover, immunoprecipitation (pull-down) analysis revealed a striking photostress-enhanced interaction of activated p300 with NF-κB-p65, thus favoring acetylation of the latter [Figure 4C]^[59]^. Another striking finding of this study is that Sirtuin-1 (Sirt1), a Class-III deacetylase that modulates gene expression by catalyzing acetyl group removal^[66]^, was strongly downregulated in photostressed U87 cells [Figure 4D], whereas a homolog, Sirt2, was unaffected^[59]^. Along with these effects, there was a striking post-hν upregulation of type-4 bromodomain and extra-terminal domain (BET) protein (Brd4), an epigenetic "reader” and transcriptional co-activator for various stress-responding genes^[63]^ In contrast, Brd2 (a paralog of Brd4) was unaffected, providing another example of signaling specificity in this system. Brd4-regulated expression of stress proteins such as E-selectin and IL-8 was first demonstrated for lung cancer cells^[67]^, but our studies were the first to link Brd4 to iNOS expression in glioblastoma cells^[59,63]^. Looking at other events upstream of iNOS transcription, Fahey *et al.*^[59]^ found that p65-acK310 formation in photostressed U87 cells was dependent on phosphorylation-activation of PI3K. This stimulated phosphorylation-activation of protein kinase B (Akt) which, in turn, depended on activation of phosphoinositide-dependent kinase-1 (PDK1). PI3K/Akt-mediated signaling is known to play a central role in cancer cell survival and proliferation^[67]^. A specific PI3K inhibitor (LY294002) prevented p300 activation as well as iNOS upregulation after an ALA/light challenge, thereby linking the iNOS response to upstream events set in motion by photodynamic stress. Evidence for another key upstream event was also obtained, *viz.* oxidative inactivation of tumor suppressor PTEN, which would have fostered PI3K/Akt activation via elevation of phosphatidylinositol triphosphate (PIP_3_) level^[59]^. Taken together, the above findings, which are depicted schematically in Figure 5, reveal a well-coordinated stress signaling network leading ultimately to iNOS/NO induction and a pro-survival/ expansion outcome. Other pro-survival effectors, e.g., COX-2, Survivin, and S100A4, are upregulated by photostress similar to iNOS/NO^[56]^, but it is not yet clear whether this occurs independently of NO or results from downstream signaling by NO^[19,20]^.

How ALA/light-induced NO can elicit photokilling resistance or greater aggressiveness of surviving cells is a question of ongoing interest. Since NO does not scavenge ^1^O_2_^[68]^, this has been ruled out as a possible cytoprotective mechanism, leaving open the possibility of downstream species scavenging. Studies by Niziolek *et al.*^[68,69]^ revealed that NO from the chemical donor spermine-NONOate (SPNO) could suppress PpIX-sensitized (^1^O_2_-initiated) lipid peroxidation in model membranes and also breast cancer cells. In the latter case, ALA-induced PpIX was allowed to diffuse from mitochondria to plasma membrane before cell irradiation in the absence *vs.* presence of SPNO. Irradiated cells died mainly by membrane-breaching necrosis and NO protected against this by acting as a chain-breaking antioxidant, as was observed previously by Rubbo *et al.*^[70]^, using a non-photodynamic model system. Niziolek *et al.*^[68,69]^ deduced that, in their system, NO acted by intercepting chain-carrying lipid-derived radicals, i.e., $LOO^{\cdot }/LO^{\cdot }$, thereby protecting cells against necrosis due to free radical-mediated membrane damage. There is no evidence yet as to whether endogenous NO can act similarly on peroxidation of mitochondrial membrane lipids sensitized by ALA-induced PpIX. Such peroxidation is highly likely, given that PpIX accumulates initially in mitochondrial membranes^[39,40]^. Whereas chain breaking by NO occurs via an irreversible chemical reaction, reversible NO reactions can also take place and fall into the signaling category. A well-known example is protein S-nitrosation, i.e., reaction of specialized cysteine thiol groups with NO (or more likely NO-derived N_2_O_3_) to give S-nitroso (SNO) adducts^[57]^. Except for a conference report in 2002^[71]^, no solid evidence for SNO formation in the context of PDT has been reported thus far. In contrast, several effector proteins have been reported to undergo S-nitrosation in non-photodynamic systems, including: (1) mitogen-activated protein kinases (MAPKs) such as ASK-1 and Jun-N-terminal kinase (JNK), whose pro-apoptotic activities are inhibited^[72]^; (2) caspase-9, whose pro-apoptotic activation or activity is inhibited^[73]^; (3) anti-apoptotic Bcl-2, whose ubiquitination and proteosomal degradation are inhibited^[74]^; and (4) anti-apoptotic MAPK phosphatase-1 (MKP-1), whose proteosomal degradation is also inhibited^[75]^. Protein S-nitrosation can be monitored by mass spectrometry, but analysis is complex and the modification is often transient due to thioredoxin-mediated denitrosation^[76]^. In the case of PDT, the latter could occur at some point after photostress is incurred, so optimal timing of cell or tissue analysis after irradiation poses a challenge.

## BYSTANDER EFFECTS OF PDT-UPREGULATED INOS/NO

Most advanced tumors, including glioblastomas, have a limited vascular supply, and, because of this, not all tumor cells will be uniformly accessed by an active PS or pro-PS such as ALA. Moreover, during subsequent irradiation, some cells will be less exposed than others due to light field limits, variable tumor geometry, and other complex factors. Thus, it is conceivable that cells experiencing the greatest photodynamic stress might respond to it by sending signals to non- or weakly-stressed neighboring cells, i.e., bystanders. Such a phenomenon is well documented for cancer cells exposed to ionizing radiation (e.g., X-rays and γ-rays), and various signaling mediators have been described, including NO^[77,78]^. To determine whether bystander effects might also apply to PDT, Bazak *et al.*^[79,80]^ developed a novel approach involving impermeable silicone rings to initially separate targeted cells (ALA/light-treated, outside rings) from non-targeted bystanders (light-only, inside rings) on a large culture dish. At some interval (e.g., 2 h) after a given light fluence (e.g., 1 J/cm^2^) from an LED source, rings are removed and responses in both cell compartments are monitored during subsequent dark incubation, e.g., iNOS/NO levels and proliferation/ migration rates. Initial experiments with human prostate carcinoma PC3 cells revealed not only an expected boost in iNOS/NO level and growth/migration rate of targeted cells, but similar responses in non-stressed bystander cells^[79]^. Although the latter responses were more moderate, they were inhibited by 1400W, cPTIO, or knockdown of targeted cell iNOS, implying that NO produced by targeted cell iNOS was responsible for the bystander effects. Use of a NO fluorescence probe (DAF-FM-DA) provided more direct evidence for this^[79]^. Conditioned medium from targeted cells did not induce bystander effects, suggesting that short-lived, continuously generated NO was solely responsible. In addition to iNOS, several other pro-tumor effectors were upregulated in PC3 bystanders, including Akt, ERK1/2, and COX-2^[79]^. In more recent studies, similar NO-mediated bystander effects were observed using glioblastoma U87 cells, and they were compared with those obtained with prostate PC3, breast MDA-MB-231, and melanoma BLM cells. After ALA treatment, irradiation conditions were adjusted to produce the same cell kill for all four types (∼25%), thus allowing clear conclusions to be made about NO-elicited resistance. Under these conditions, bystander proliferation and migration rates increased with extent of iNOS upregulation in surviving targeted cells in the following order: BLM < U87 < MDA-MB-231 < PC3^[80]^. Thus, targeted cells with the greatest iNOS/ NO induction after an ALA/hν challenge elicited the greatest increases in bystander aggressiveness. These findings suggest that a NO-based “relay” process is set in motion by photodynamic stress. In this process, NO overproduced by targeted cells (e.g., U87 or U251) diffuses to non-stressed bystanders and induces iNOS/NO there, thus beginning a NO “feed-forward” process that propagates through the bystander population. Whereas photodynamic stress activates NF-κB and thence iNOS transcription in targeted cells, the transcription factor responsible for NO-initiated iNOS induction in bystander cells has not yet been defined. If occurring in an actual tumor, e.g., GBM, after a PDT challenge, NO-mediated bystander effects might stimulate tumor growth and metastatic expansion. While this unfortunate possibility is well recognized in connection with therapeutic ionizing radiation^[78]^, it is still not so with regard to PDT for any solid malignancies, including glioblastomas. As discussed in the next section, these negative effects of NO from targeted cells could be attenuated by pharmacologic interventions aimed at either inhibiting iNOS enzymatic activity or iNOS transcription. This would be expected to increase the overall anti-tumor efficacy of PDT at the clinical level.

## PHARMACOLOGIC MITIGATION OF NITRIC OXIDE'S ANTI-PDT EFFECTS

Although not yet tested in the clinic, it is likely, based on evidence presented above, that inhibiting iNOS activity or expression would significantly improve PDT outcomes against glioblastoma and other solid tumors. At least two iNOS activity inhibitors, L-NIL and GW274150, have already been tested in clinical trials, but these were unrelated to cancer or PDT^[81,82]^. Instead, both agents were tested for relieving asthmatic inflammation and, importantly, neither one had any negative side effects. As indicated above, GW274140 significantly improved PDT efficacy in a human breast tumor xenograft model^[58]^, suggesting that this inhibitor would be a good test adjuvant for clinical PDT against gliomas and other solid tumors. As already discussed, iNOS transcription in glioblastoma cells is regulated by NF-κB subunit p65, which is activated by p300-catalyzed acetylation of lysine-310^[59]^. Knowing this and that (1) Brd4 is a necessary co-activator of iNOS transcription; (2) Brd4 is increasingly upregulated by photostress; (3) p65 is increasingly K310-acetylated by photostress; and (4) that the latter promotes Brd4 interaction with p65^[59,63]^, we asked how the latter response might be suppressed in order to reduce iNOS upregulation in ALA/light-challenged glioblastoma cells. Bromo- and extra-terminal domain (BET) proteins act as epigenetic “readers” of acetylated lysine residues on histones and transcription factors, thereby co-regulating gene transcription at promoter sites^[83]^. BET protein inhibitors such as JQ1 and OTX015 were recently introduced as powerful new means of suppressing tumor development and progression at the transcriptional level^[84,85]^. These inhibitors function by binding to BET domains on Brd4 and other BET proteins, thereby preventing interaction with acK groups on transcription factors (e.g., p65-acK310) or on histones^[85]^. When tested on ALA/light-treated U87 cells, JQ1 at a minimally cytotoxic concentration: (1) increased cell killing synergistically compared with photostress alone; (2) strongly inhibited Brd4 binding to p65-acK310; (3) greatly reduced iNOS/NO upregulation after irradiation; and (4) nearly abolished the hyper-aggressiveness of cells that could withstand the ALA/light challenge^[63]^. One other striking observation in this study is that the concentration of JQ1 used (∼0.3 μM) was far below that of 1400W capable of producing similar effects. Another glioblastoma line, U251 cells, responded similarly to JQ1 after being photostressed^[63]^. In addition to iNOS, several other NF-κB-regulated proteins were affected by photostress in U87 cells, including pro-survival Bcl-xL and Survivin, which were upregulated, and tumor suppressor p21, which was downregulated^[63]^. Each of these photostress responses, similar to iNOS upregulation, was strongly suppressed by JQ1, thereby promoting cell photokilling^[63]^. Although Bcl-xL and Survivin transcription may have been directly affected by JQ1, an indirect iNOS/NO-mediated effect was also possible, since NO is known to modulate expression of these effector proteins^[19,20]^. Thus, in at least these two cases, JQ1 could have acted directly by preventing Brd4 binding at promoter sites and/or indirectly by inhibiting iNOS expression. In any event, JQ1 inhibition of iNOS transcription appeared to play the major role in improving the efficacy of glioblastoma cell photokilling. It is clear, therefore, that JQ1 would make a highly promising PDT adjuvant, particularly since it has already been used successfully with other anti-cancer therapies. In the case of glioblastoma, for example, JQ1 has been reported to synergize with temozolomide in cytotoxicity at the in *vitro* as well as *in vivo* level^[86]^. We anticipate that JQ1 or some other BET inhibitor will act similarly when used in combination with PDT in glioma animal models and eventually glioma patients.

## CONCLUSIONS

The many attractive features of ALA-PDT, including tumor site specificity, non-toxicity of components individually (ALA-induced PpIX, light, and O_2_), and its demonstrated efficacy on difficult tumors such as glioblastomas, make it an appealing therapy for these malignancies^[8–10,41]^. An added advantage of using ALA is that tumor-localized PpIX can be employed for fluorescence-guided surgery (FGS), which is often followed up by PDT to eradicate any residual cells^[43]^. It is now well established that cells in many tumors, including gliomas, exploit low-level NO to avoid apoptosis, stimulate proliferation and migration, and resist radio- or chemotherapy^[19–21]^. As pointed out above, such NO can also impose a strong resistance to PDT. The NO can derive from tumor cells themselves, although proximal vascular cells (macrophages, fibroblasts, and endothelial cells) may contribute. The *in vitro* and *in vivo* studies described in this review are unique in demonstrating that endogenous iNOS/NO in many tumor cells, including glioblastomas, plays a major role not only in PDT resistance, but also enhanced aggressiveness of surviving cells and non-targeted bystanders. Although both basal and photostress-induced iNOS might be implicated in these responses, there is now solid evidence that induced enzyme plays a preponderant role in several cancer types^[13]^. This evidence is unprecedented because most therapy-based studies up to now have considered only pre-existing iNOS/NO and not the possibility of overexpression due to the treatment itself. Concerns about a more aggressive (proliferative and migratory/invasive) phenotype of PDT-surviving cells could be mitigated by turning to pharmacologic inhibitors of iNOS enzymatic activity or iNOS transcription. We suggest possible candidates in each of these categories, emphasizing the greater advantages of those in the latter category, i.e., BET inhibitors.
